# Observations on a Novel Bacterial Pathogen of Root-Knot Nematodes (*Meloidogyne* spp.)

**DOI:** 10.3390/pathogens10101226

**Published:** 2021-09-22

**Authors:** Aurelio Ciancio

**Affiliations:** Istituto per la Protezione Sostenibile delle Piante, Consiglio Nazionale delle Ricerche, 70126 Bari, Italy; aurelio.ciancio@ipsp.cnr.it

**Keywords:** bacteriosis, disease, infection, juvenile, parasitism, septa

## Abstract

A novel Gram-negative pathogenic bacterium (BN) was discovered in second-stage juveniles (J2) of root-knot nematodes (RKN, *Meloidogyne* spp.). Mature bacteria showed a peculiar rod morphology characterized by four cells sequentially joined at septa. Mature rods measured 4–5 × 0.5–0.6 μm and were characterized by the emptying and tapering of both apical cells. The data showed an electron-dense external matrix forming a coating capsule involved in host attachment. The rods were not motile and packed in parallel inside the J2 body. After J2 penetration by adhering, germinating cells, the bacterium proliferated until the host body content was completely digested, producing a lethal disease. Parasitized hosts were recognized using light microscopy by a pale creamy-brown color assumed at parasitism completion. At death, the whole nematode body was filled with cells and only a few sclerotized esophageal structures (i.e., stylet, median bulb) remained visible. The BN cells were quickly released at host body rupture, suggesting that J2 infection occurs through passive adhesion of cells dispersed in soil. The bacterium appeared fastidious, as attempts to obtain pure cultures on common nutritive media failed.

## 1. Introduction

Nematodes and bacteria have a long history of coevolutionary links, as shown by the number of highly specialized species reported in strict association with nematodes from soil or marine environments [1,2,3]. Progress in genome and metagenome studies showed an increased number of associations based on a range of trophic relationships varying from parasitism to different types of symbiosis [2,3]. Such a broad diversity of taxa is expected to increase further as far as studies on the nematode-bacteria associations proceed considering the diversification of the phylum Nematoda, the richness of environmental niches colonized, and the specialized metabolic interactions established [1,2,3,4].

The most common bacterial group often reported in, but not limited to, plant parasitic nematodes includes the Gram-positive, spore-forming obligate parasites of the genus *Pasteuria* (Firmicutes: Pasteuriaceae) [5,6]. Further interactions include *Corynebacterium* spp. associated to foliar nematodes and *Streptomyces costaricanus*, a biocontrol agent of root-knot nematodes (*Meloidogyne* spp.), isolated from a suppressive soil [7,8,9]. Several *Bacillus* and *Pseudomonas* spp. and/or strains have also been described or reported as biocontrol agents of plant parasitic nematodes [10,11,12,13,14] or free-living species [15].

Many interactions with bacteria have also been discovered in animal parasitic and marine nematodes, including, i.e., intestine-inhabiting bacteria causing cuticular lesions in horse-parasitic ascarid and strongylid species [16,17]. *Moraxella osloensis*, a bacterium associated with the slug-parasitic nematode *Phasmarhabditis hermaphrodita*, was found to be lethal to the slug and necessary for the nematode’s biological activity [18]. Species from genera *Xenorhabdus* and *Photorhabdus* are well-known endosymbionts of the entomopathogenic nematodes *Steinernema* and *Heterorhabditis*, respectively, playing a fundamental role in their lifecycle [1,19].

Intracellular endosymbionts have been repeatedly reported in cyst nematodes [20,21,22], whereas endosymbionts related to *Wolbachia* spp. have been found in filarial and plant parasitic nematodes as well [3,23,24,25]. Nematode endosymbiotic associations include “*Candidatus Paenicardinium*” from *Heterodera glycines* [26]. Finally, a further lineage of vertically transmitted endosymbionts belonging to the phylum Verrucomicrobia was described in *Xiphinema* spp., characterized by a nutritional mutualistic relationship with the host [27,28].

Root-knot nematodes (RKN, *Meloidogyne* spp.) are damaging and economically important plant pests, distributed all over the world and endemic in many regions on horticultural or perennial crops. In some populations sampled in Southern Italy at high juvenile (J2) densities (in the order of 10^3^ J2/100 mL soil^−1^), a novel undescribed Gram-negative bacterial parasite (herein termed BN = “*Bacterium nematophagum*”) was observed producing a previously unknown disease lethal for nematodes. The parasite displayed a particular morphology and appeared as a novel rhizosphere bacterium. Different BN strains characterized by a unique, specific morphology, were consistently observed in J2 from distinct populations of *M. javanica* or *M. incognita* proceeding from Apulia or Sicily.

The pathology induced and the bacterium itself did not match any disease or species previously reported in or associated with RKN. The diseased hosts were easily recognized with light microscopy because of a pale creamy-brown color they assumed after infection. This peculiar condition is also new and was observed only in the dead specimens, from which several BN cells were released after body rupture.

Due to the interest and potentialities related to new undescribed bacterial species and because of the increasing attention dedicated today to biological control agents of plant pests, this novel host–parasite association was investigated. The first observations and the data showing BN morphology, ultrastructure, and host parasitism are presented and discussed herein.

## 2. Results

**Figure 1:**
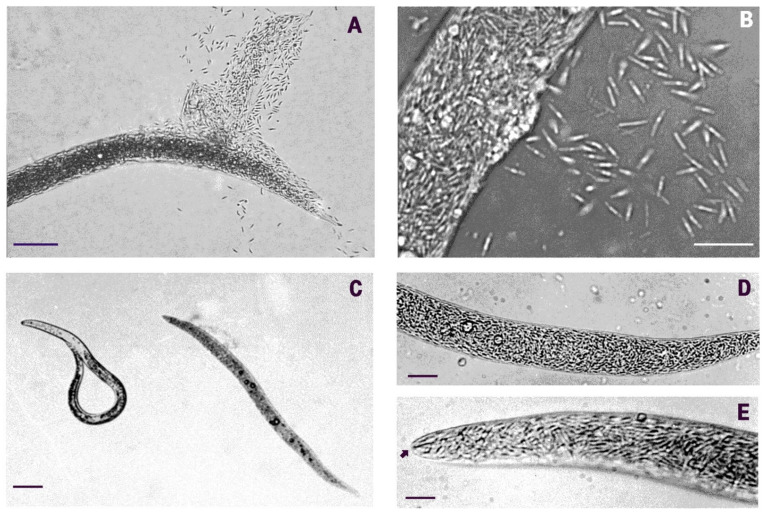
LM images of the nematode-parasitic bacterium (BN) in juveniles of (**A**) *Meloidogyne javanica*, population: BN-RAG; and (**B**) *M. incognita*, population: BN-MOL. Parasitism (**C**) was revealed by a creamy-brown color of diseased hosts (a parasitized *M. javanica* J2 on the right, a healthy nematode on the left; population: BN-LEV). (**D**,**E**) The nematodes were completely filled with bacterial cells (population: BN-LEV). (**E**) At the end of the infection, only the stylet and few other cuticular structures remained visible under LM (arrow). Scale bars: (**A**): 50 μm; (**B**): 12 μm; (**C**): 40 μm; (**D**): 14 μm; (**E**): 6 μm.

Nematodes showing symptoms of BN-induced infection were found among the second-stage juveniles (J2) of four root-knot nematode populations, with the highest prevalence levels (up to 50%) observed by late summer. The dead specimens lost any body turgor and released thousands of bacterial cells after body collapse or when ruptured with gentle pressure on agar or under a cover glass (Figure 1A,B). Light microscopy (LM) examinations showed that the pathology was always associated to a color switch of parasitized hosts, which turned from pale brown to creamy as the disease progressed. This feature allowed the recognition of infected specimens during routine J2 counts. Moreover, diseased nematodes often showed aligned oil droplets within their bodies (Figure 1C,D).

**Figure 2:**
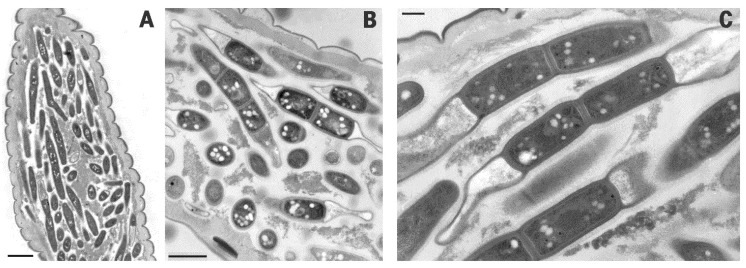
Transmission electron microscopy (TEM) images of mature BN cells (population: BN-LEV) filling the whole body of *M. javanica* J2 (**A**). The cells appeared as septate rods rich in reserve granules (**B**,**C**). The peculiar morphology of the BN resulted from the persistence of division septa between cells surrounded by an external coat and the flattening of both apical cells (**C**). Scale bars: (**A**) = 3 μm; (**B**) = 1 μm; (**C**) = 300 nm.

No internal anatomical structure of the J2 remained intact at disease completion as the whole nematode body content was digested apart from a few sclerotized parts such as the median bulb, esophagus, cuticle, and stylet (Figure 1D,E and Figure 2A). When smeared on slides from squashed J2, BN cells stained Gram-negative, as did all the bacterial cells with the same morphology remaining inside the squashed specimens.

**Figure 3:**
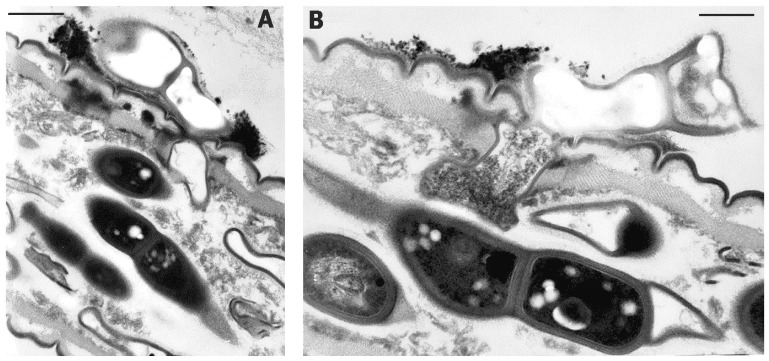
TEM images of the nematode infection process showing germination of BN cells adhering to the host cuticle (population: BN-LEV). After adhesion to the host, a lateral growing cell penetrated the nematode body through its cuticle and hypodermis layers (**A**,**B**). Scale bars: (**A**) = 650 nm; (**B**) = 425 nm.

**Figure 4:**
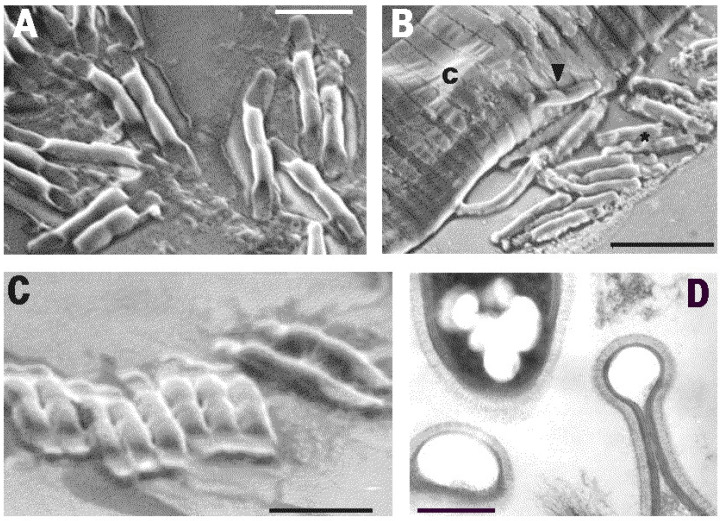
Cells of BN from the collapsed *M. incognita* J2 (population: BN-LEV), showing the typical morphology of the bacterium (**A**). A SEM image of the cells released (**A**) or adhering (**B**) (arrowhead) to the host nematode cuticle (c). After the release, the BN cells appeared frequently packed in rows (**C**). A TEM image showing the bacterium’s apical tapering, giving a hairpin-like appearance (**D**). Cell wall section shows the outer membrane, the coat, and an external thin layer of adhesive fibers. Scale bar (**A**): 2 μm; (**B**): 4.6 μm; (**C**): 2.7 μm; (**D**): 375 nm.

TEM examinations of BN cells from the diseased specimens showed the bacterium as a segmented rod, confirming previous LM observations (Figure 2B,C). Cell walls in the mature cells remained joined after cell division. They were not observed during the first vegetative stages, in which the BN cells appeared as elongated, electron dark rods (Figure 2A). The mature segmented rods measured 4–5 μm in length and 0.5–0.6 μm in width (Figure 3 and Figure 4).

TEM and SEM observations showed that BN cells adhered to the host nematode cuticle (Figure 3 and Figure 4B). Serial sections showed that, after adhesion, the parasite germinated and eventually penetrated the host cuticle and hypodermis layers through a lateral branch or budding, reaching the internal host tissues (Figure 3A,B). TEM images showed that these stages were associated with the multiplication phase, spreading the infection within the host and filling its whole body with septate cells at different maturing stages (Figure 2A). TEM examinations confirmed total body digestion as observed with LM, with the exclusion of the more external cuticular layers (Figure 1E and Figure 2A–C). The induction of germination was not related to any host metabolic activity or motility since it was also observed in the parasitized or already killed nematodes (Figure 3).

TEM sections showed a complex cell wall, typical of Gram-negative bacteria. The inner BN cytoplasmic membrane was separated by a periplasmic space from an 8–10 nm wide electron-dense peptidoglycan layer (Figure 3B and Figure 4D). After this layer, a 30–40 nm thick and layered outer membrane was present, covered by a further electron dense coat, 12–20 nm thick. An additional external layer of O-side chains forming a matrix of adhesive fibers 50–62 nm thick followed the coat. O-side chains show a thin electron dark peripheric contour, 12–15 nm thick (Figure 4D).

The coat and fiber layers enveloped the whole rod, which at maturity was mostly composed of four attached cells. These layers were also visible in immature, unsegmented rods, but were not found in the wall of the germinating pegs, which appeared simpler and thinner (Figure 3A,B). At the round, blunt apex of the mature terminal cells, either the outer membrane or the coat gradually tapered to form a progressively thinner wall, 18 nm thick (Figure 4D). The outer surface of the rods attached to the nematode cuticle frequently showed adherent electron dark aggregates of clay-like microparticles or minerals, suggesting the presence of superficial electric charges (Figure 3A,B).

BN rods were characterized by a unique shape and morphology resulting from the symmetric emptying and flattening of both terminal cells or compartments (Figure 2B,C and Figure 3A,B). Empty apical cells were mainly found in mature rods at the end of the parasitic stage or in the nematodes already dead and filled with rods (Figure 2C and Figure 4D). The terminal cells appeared collapsed and flattened due to the loss of their cytoplasmic content. Their opposite walls often approached in direct contact, originating a typical hairpin-like contour visible in longitudinal sections and also observed with SEM (Figure 4A–D). This morphology was also visible with LM at highest magnifications when BN smears from squashed nematodes from any population were examined in temporary water mounts (Figure 1B). This peculiar shape of the bacterium was consistently observed among the different nematode and BN populations examined and appeared useful in the identification and visual diagnosis of the disease. Under LM, the rods presented with a refractile central region corresponding to the inner cell pairs filled with the cytoplasm, with an opaque peripheric portion corresponding to the terminal flat ends (Figure 1B). The combination of cell shape, symmetry, and apical flattening gave rise to a particular and distinctive morphology of the rods characterized by a typical bilateral flute-like appearance (Figure 2B and Figure 4A–C).

BN cells multiplied through a centripetal formation of a transverse septum (data not shown). Lateral budding was observed in immature forms or at germination during host infection (Figure 2A and Figure 3B). At cell wall formation, the external coat layer of the mother cell was kept, filling the inner space between the newly formed opposite walls, separating the walls of two adjacent cells and acting as a cement between them (Figure 2C and Figure 3B). At septum completion, the cells remained embedded and covered by the coat and adhesive matrix (Figure 3B).

Several electron transparent granules similar to poly-β-hydroxybutyrate inclusions were frequently observed in the BN cytoplasm, as well as in immature rods (Figure 2B,C, Figure 3A,B and Figure 4D). Further cell organelles included electron dark vesicles of variable sizes, mainly found scattered in the inner cell pairs, nucleoids, and ribosomes (Figure 3B).

TEM and SEM examinations did not show any flagella or flagella-like filaments at any stage of BN multiplication. Furthermore, no direct or gliding motility was recorded when examining clusters of BN rods dispersed on water agar or CMA with LM at weekly intervals. The bacterium appeared fastidious in nature, and any attempt to obtain pure cultures or induce its multiplication on the nutritive media tested failed.

Prevalence data from the *M. javanica* population sampled at Pedalino (Ragusa) showed that the parasite was almost undetectable when J2 numbers were low, increasing its frequency in a density-dependent manner as the nematode population increased.

## 3. Discussion

The large amount of metagenome data accumulated thus far has shown that a large portion of the bacterial diversity present in the environment, including agricultural soils, remains still unknown or not classified [29]. This consideration is supported by studies from a wide range of microenvironments, suggesting that obligate and fastidious metabolism characterizes a significant fraction of the total bacterial diversity occurring in nature [30]. The preliminary data shown herein, although limited to the BN morphology and biology, suggest that the bacterium represents a novel Gram-negative species specialized in obligate nematode parasitism. No record about similar microorganisms or pathologies was found in the literature, whereas no correspondence could be directly assigned between the BN and other bacteria known as associated with nematodes.

Filamentous bacteria have been reported in different nematodes, including marine species [31] or from cyathostomins (Nematoda: Strongylidae) parasitic in African equids [32]. The species inhabiting the cuticle of the marine nematode *Eubostrichus dianae* showed a complex community of distinct bacterial taxa, as shown by 16S rDNA sequencing [31]. The general morphology of these species, however, was distinct from that of BN. The bacteria reported in association with cyathostomid nematodes included septate and very long (>500 μm) *Arthromitus*-like species found attached to the nematodes’ natural openings. They also appeared morphologically distinct from the BN, apart from some similarities in the cell wall structure [32]. The cyathostomid microbial community also included other smaller, blunt-ended organisms. The data available on their biology and ultrastructure were, however, not sufficient to establish a relationship with the BN, if any [33]. Finally, the BN differed from non-pathogenic, segmented filamentous bacteria with blunt ends attached externally to the cuticle in the anal or vulvar regions or the reproductive tract of cyathostomins from the hindgut of zebras. The diverse community of bacteria reported presented with different and concomitant taxa, some of which were characterized by a Gram-positive reaction, greater dimensions than that of the BN, production of septa, and/or presence of *Clostridium*-like endospores [34].

The observations reported suggest that the BN is a highly specialized and obligate rhizosphere bacterium dependent for growth on the host metabolism. The in vitro reproduction of the pathology and the validation of Koch’s postulates, however, were limited by the absence of available BN-culturing media and by the low numbers of cells that it was possible to handle from crushed hosts. The association of the bacterial parasite with the disease was assessed anyway. Together with the consistent isolation of BN cells from the J2 cadavers with the creamy color typical of the disease, the host-parasite association provided support to consider the first and second of Koch’s postulates to be at least satisfied. Adhesion of BN cells (proceeding from disrupted nematodes) to RKN J2 exposed in vitro was also assessed (data not shown).

The disease may have practical implications as the BN appeared to be a specialized antagonist, endemic in *Meloidogyne*-infested soils, as indicated by the density-dependent host relationship. Because of J2 parasitism, the bacterium holds a potential in RKN biocontrol, as it kills the nematodes before root penetration and any tissue damage occur. It may hence be added to the other microbial components of the soil microflora that contribute to natural regulation of RKN, with a possible reference to soil suppressiveness [5,6,35].

The systematic position of the BN remains unclear, pending culturing and 16S rRNA ribosomal gene sequencing. Attempts to produce unambiguous PCR products for the 16S gene consistent among different populations and experiments failed (data not shown). Cell wall organization appeared typical of Gram-negative species [36] and more complex than that of other nematode endosymbionts, i.e., Verrucomicrobia [27]. Some similarities with *Rhizobium* suggested a first putative assignment to proteobacteria (R. Favre, personal communication). The thick external coat covering the whole rod appeared, however, as a specific characteristic involved in host adhesion and likely responsible for the host–parasite specificity.

Some lifecycle similarities with Gram-positive parasites such as *Pasteuria* spp. arose from a number of biological processes including propagule adhesion, host penetration, outgrowth, and release from cadavers. This behavior, however, appears to be the outcome of convergent evolution, mainly determined by the host nematode anatomy and structural organization of the body. Differing from *Pasteuria*, no sporulating stage or endospore was observed in the BN at any stage.

Although of limited value in bacterial taxonomy, the peculiar morphology exhibited by the BN is a phenotypic trait likely reflecting functional adaptation to host parasitism. Flat and empty apical cells did not appear as artefacts as they were consistently observed in several replicates at high resolution with LM, TEM, or SEM. The blunt rod apex pairs likely have a role in keeping firm cell adhesion to the J2 cuticle and, together with the matrix of the coating capsule, appeared sufficiently strong to hold the central infective cells in place during their subsequent activation and germination.

## 4. Materials and Methods

### 4.1. Sampling of the Host Populations

Cells of the BN were initially observed in Apulia in association with a population of *M. javanica* parasitizing carnations in a greenhouse at Leverano (bacterial population identified as BN-LEV). Further BN populations were observed in Apulia in association to J2 from an *M. incognita* population parasitizing tomatoes at Molfetta (population: BN-MOL), an *M. javanica* population attacking tomatoes at Canosa and, in Sicily, from a further *M. incognita* population parasitizing almonds at Pedalino (Ragusa, population: BN-RAG). Nematode and BN populations were maintained in the greenhouse on cherry tomatoes at 25–28 °C, in distinct pots with the original soils.

### 4.2. Light Microscopy (LM)

Nematodes were extracted from soil with Cobb’s sieving and decanting technique using a set of 720 μm and 45 μm sieves [37]. The diseased specimens were identified during J2 counts in a 1 mL Hawksley chamber under a Leitz Orthoplan light microscope at 40×. Non-motile J2 showing the color changes associated to the BN disease were gently recovered from the chamber using an eyelash glued to a needle tip. The specimens collected this way were then stored in tap water in a watch glass and/or directly placed on a glass slide in a temporary water mount for inspection at 400–1000×. Digital images were captured using a Hamamatsu C 5810 CCD camera mounted on the microscope and subsequently digitally processed. Images of clustered BN cells on CMA were captured with the CCD and compared at one-week intervals to check mobility and division. Plates with other media were also periodically examined for BN proliferation. Gram staining was performed on the cells released from the nematodes ruptured on a glass cover slide, fixed by fast flame exposure and stained with safranin and crystal violet (SIGMA HT90-A) [38].

### 4.3. Transmission Electron Microscopy (TEM)

The diseased *M. javanica* J2 filled with BN-LEV cells were extracted from soil as previously described and selected for TEM preparation. The nematodes were handpicked from the suspension obtained by sieving and examined in temporary water mounts with LM at 250–400× to check occurrence of the disease. The diseased specimens were then embedded in 2% agarose, fixed in the glutaraldehyde–cacodylate buffer for 3 h or overnight, post-fixed in 1% OsO_4_ in the cacodylate buffer, dehydrated in an ethanol series with 0.5% uranyl acetate at 70% ethanol, and then washed three times in 100% ethanol and propylene oxide before embedding the blocks in the Polybed resin. Serial sections 60–80 nm thick were cut with a Reichert microtome, stained with uranyl acetate and lead citrate solutions, and examined with a Philips EM 208 electron microscope at 100 kV.

### 4.4. Scanning Electron Microscopy (SEM)

The morphology of BN cells and their morphometrics were checked with SEM, examining cell clusters released from the ruptured nematodes. The diseased J2 of the *M. javanica* population from Leverano were handpicked from the sieving suspension and identified as described above. The nematodes were dehydrated with glycerol with the slow method [37] and maintained in glycerol until used. For scanning, the specimens were handpicked and mounted on a metal stub cleared of excess glycerol, cut or crushed directly on the stub to release the BN cells, and sputter-coated with gold in vacuum. The samples were then examined with a Stereoscan 360 Cambridge Instruments SEM at 3 kV [39].

### 4.5. Culturing Assays

The infected nematodes identified with LM were collected from different populations. BN cells collected from the crushed nematodes with a sterile pipette or whole infected J2 handled with an ethanol-sterilized and air-dried eyelash were used in attempts to produce BN cultures. The solid media tested were as follows: 1.5% water agar (WA), corn meal agar (CMA); Grace’s insect medium (Difco, Detroit, MI, USA) with agar (15 g/L) with or without hemin (5 mg/L); yeast extract (1 g/L) with agar (15 g/L), peptone (5 g/L), and NaCl (5 g/L), with or without lactic acid (30 mL/L). Some BN-infected nematodes were also ruptured and smeared on Petri dishes with the tested media after gentle rinsing in sterile distilled water. Petri dishes were stored at 25 °C in the dark and examined periodically for growth during the following weeks [40].

## Data Availability

Not applicable.

## References

[1] Poinar G.O., Hansen E.L. (1986). Associations between nematodes and bacteria. Helminth. Abstr. Ser. B.

[2] Bandi C., Anderson T.J., Genchi C., Blaxter M.L. (1998). Phylogeny of *Wolbachia* in filarial nematodes. Proc. R. Soc. Lond. B Biol. Sci..

[3] Brown A., Wasala S., Howe D., Peetz A.B., Zasada I.A., Denver D.R. (2016). Genomic evidence for plant-parasitic nematodes as the earliest *Wolbachia* hosts. Sci. Rep..

[4] Palomares-Rius J.E., Archidona-Yuste A., Cantalapiedra-Navarrete C., Prieto P., Castillo P. (2016). Molecular diversity of bacterial endosymbionts associated with dagger nematodes of the genus *Xiphinema* (Nematoda: Longidoridae) reveals a high degree of phylogenetic congruence with their host. Mol. Ecol..

[5] Siddiqi Z.A., Mahmood I. (1999). Role of bacteria in the management of plant parasitic nematodes: A review. Biores. Technol..

[6] Stirling G. (2014). Biological Control of Plant-Parasitic Nematodes: Soil Ecosystem Management in Sustainable Agriculture.

[7] Bird A.F., Riddle D.L. (1984). Effect of attachment of *Corynebacterium rathayi* on movement of *Anguina agrostis* larvae. Int. J. Parasitol..

[8] Taylor C.E. (1990). Nematode interactions with other pathogens. Ann. Appl. Biol..

[9] Esnard J., Potter T.L., Zuckerman B.M. (1995). *Streptomyces costaricanus* sp. nov., isolated from nematode-suppressive soil. Int. J. Syst. Bacteriol..

[10] Hackenberg C., Muehlchen A., Forge T., Vrain T. (2000). *Pseudomonas chlororaphis* strain Sm3, bacterial antagonist of *Pratylenchus penetrans*. J. Nematol..

[11] Kluepfel D.A., Nyczepir A.P., Lawrence J.E., Wechter W.P., Leverentz B. (2002). Biological control of the phytoparasitic nematode *Mesocriconema xenoplax* on peach trees. J. Nematol..

[12] Meyer S.L.F., Roberts D.P. (2002). Combinations of biocontrol agents for management of plant-parasitic nematodes and soilborne plant-pathogenic fungi. J. Nematol..

[13] Meyer S.L.F., Roberts D.P., Chitwood D.J., Carta L.K., Lumsden R.D., Mao W. (2001). Application of *Burkholderia cepacia* and *Trichoderma virens*, alone and in combinations, against *Meloidogyne incognita* on bell pepper. Nematropica.

[14] Tian H., Riggs R.D., Crippen D.L. (2000). Control of soybean cyst nematode by chitinolytic bacteria with chitin substrate. J. Nematol..

[15] Huang X.W., Niu Q.H., Zhou W., Zhang K.Q. (2005). *Bacillus nematocida* sp. nov., a novel bacterial strain with nematotoxic activity isolated from soil in Yunnan, China. Syst. Appl. Microbiol..

[16] Anderson W.R., Madden P.A., Tromba F.G. (1971). Histopathologic and bacteriologic examinations of cuticular lesions of *Ascaris suum*. J. Parasitol..

[17] Anderson W.R., Madden P.A., Colglazier M.L. (1978). Microbial flora of cuticular lesions on *Strongylus edentatus*. Proc. Helm. Soc. Wash..

[18] Tan L., Grewal P.S. (2001). Pathogenicity of *Moraxella osloensis*, a bacterium associated with the nematode *Phasmarhabditis hermaphrodita*, to the slug *Deroceras reticulatum*. Appl. Environ. Microbiol..

[19] Forst S., Nealson K. (1996). Molecular biology of the symbiotic-pathogenic bacteria *Xenorhabdus* spp. and *Photorhabdus* spp. Microbiol. Rev..

[20] Sheperd A.M., Clark S.A., Kempton A. (1973). An intracellular micro-organism associated with tissues of *Heterodera* spp. Nematologica.

[21] Endo B.Y. (1979). The ultrastructure and distribution of an intracellular bacterium-like microorganism in tissue of larvae of the soybean cyst nematode, *Heterodera glycines*. J. Ultrastr. Res..

[22] Walsh J.A., Lee D.L., Sheperd A.M. (1983). The distribution and effect of intracellular rickettsia-like micro-organisms infecting adult males of the potato cyst-nematode *Globodera rostochiensis*. Nematologica.

[23] Ferri E., Bain O., Barbuto M., Martin C., Lo N., Uni S., Landmann F., Baccei S.G., Guerrero R., Lima S.D.S. (2011). New insights into the evolution of *Wolbachia* infections in filarial nematodes inferred from a large range of screened species. PLoS ONE.

[24] Wasala S.K., Brown A.M.V., Kang J., Howe D.K., Peetz A.B., Zasada I.A., Denver D.R. (2019). Variable abundance and distribution of *Wolbachia* and *Cardinium* endosymbionts in plant-parasitic nematode field populations. Front. Microbiol..

[25] Sironi M., Bandi C., Sacchi L., Di Sacco B., Damiani G., Genchi C. (1995). Molecular evidence for a close relative of the arthropod endosymbiont *Wolbachia* in a filarial worm. Mol. Biochem. Parasitol..

[26] Noel G.R., Atibalentja N. (2006). “*Candidatus* Paenicardinium endonii” an endosymbiont of the plant-parasitic nematode *Heterodera glycines* (Nemata: Tylenchida), affiliated to the phylum Bacteroidetes. Int. J. Syst. Evol. Microbiol..

[27] Vandekerckhove T.T.M., Willems A., Gillis M., Coomans A. (2000). Occurrence of novel verrucomicrobial species, endosymbiotic and associated with parthenogenesis in *Xiphinema americanum*-group species (Nematoda, Longidoridae). Int. J. Syst. Evol. Microbiol..

[28] Brown A.M.V., Howe D.K., Wasala S.K., Peetz A.B., Zasada I.A., Denver D.R. (2015). Comparative genomics of a plant-parasitic nematode endosymbiont suggest a role in nutritional symbiosis. Genome Biol. Evol..

[29] Kellenberger E. (2001). Exploring the unknown: The silent revolution of microbiology. EMBO Rep..

[30] Overmann J., Abt B., Sikorski J. (2017). Present and future of culturing bacteria. Ann. Rev. Microbiol..

[31] Polz M.F., Harbison C., Cavanaugh C.M. (1999). Diversity and heterogeneity of epibiotic bacterial communities on the marine nematode *Eubostrichus dianae*. Appl. Environ. Microbiol..

[32] Els H.J., Krecek R.C. (1993). Developmental stages of a smooth-walled filamentous bacterium associated with equine Cyathostomes. J. Helm. Soc. Wash..

[33] Krecek R.C., Sayre R.M., Els H.J., Van Niekerk J.P., Malan F.S. (1987). Fine structure of a bacterial community associated with chyatostomes (Nematoda: Strongylidae) of zebras. Proc. Helm. Soc. Wash..

[34] Mackie R.I., Krecek R.C., Els H.J., Van Niekerk J.P., Kirschner L.M., Baecker A.A.W. (1989). Characterization of the microbial community colonizing the anal vulvar pores of helminths from the hindgut of zebras. Appl. Environ. Microbiol..

[35] Yin B., Valinsky L., Gao X., Becker O., Borneman J. (2003). Bacterial rRNA genes associated with soil suppressiveness against the plant-parasitic nematode *Heterodera schactii*. Appl. Environ. Microbiol..

[36] Beveridge T.J. (1999). Structures of Gram-negative cell walls and their derived membrane vesicles. J. Bacteriol..

[37] Southey J.F. (1970). Laboratory Methods for Work with Plant and Soil Nematodes.

[38] Girard H., Rougieux R. (1967). Techniques de Microbiologie Agricole.

[39] Sher S.A., Bell A.H. (1975). Scanning electron micrographs of the anterior region of some species of Tylenchoidea (Tylenchida: Nematoda). J. Nematol..

[40] Leadbetter J.R. (2003). Cultivation of recalcitrant microbes: Cells are alive, well and revealing their secrets in the 21st century laboratory. Curr. Opin. Microbiol..

